# Deep temporal models and active inference

**DOI:** 10.1016/j.neubiorev.2017.04.009

**Published:** 2017-06

**Authors:** Karl J. Friston, Richard Rosch, Thomas Parr, Cathy Price, Howard Bowman

**Affiliations:** a Wellcome Trust Centre for Neuroimaging, Institute of Neurology, University College London, WC1N 3BG, UK; b Centre for Cognitive Neuroscience and Cognitive Systems and the School of Computing, University of Kent at Canterbury, Canterbury, Kent, CT2 7NF, UK; c School of Psychology, University of Birmingham, Edgbaston, Birmingham B15 2TT, UK

**Keywords:** Active inference, Bayesian, Hierarchical, Reading, Violation, Free energy, P300, MMN

## Abstract

How do we navigate a deeply structured world? Why are you reading this sentence first – and did you actually look at the fifth word? This review offers some answers by appealing to active inference based on deep temporal models. It builds on previous formulations of active inference to simulate behavioural and electrophysiological responses under hierarchical generative models of state transitions. Inverting these models corresponds to sequential inference, such that the state at any hierarchical level entails a sequence of transitions in the level below. The deep temporal aspect of these models means that evidence is accumulated over nested time scales, enabling inferences about narratives (i.e., temporal scenes). We illustrate this behaviour with Bayesian belief updating – and neuronal process theories – to simulate the epistemic foraging seen in reading. These simulations reproduce perisaccadic delay period activity and local field potentials seen empirically. Finally, we exploit the deep structure of these models to simulate responses to local (e.g., font type) and global (e.g., semantic) violations; reproducing mismatch negativity and P300 responses respectively.

## Highlights

• Active inference provides a principled account of epistemic behaviour.
• Active inference rests upon hierarchical or deep generative models.
• Deep generative models of state transitions embody nested temporal structure.
• Reading can be simulated via active inference with deep models.
• These simulations appear to have a high degree of biological plausibility.

## 1 Introduction

In recent years, we have applied the free energy principle to generative models of worlds that can be described in terms of discrete states in an attempt to understand the embodied Bayesian brain. The resulting active inference scheme (for Markov decision processes) has been applied in a variety of domains (see Table 1). This paper takes active inference to the next level and considers hierarchical models with deep temporal structure (George and Hawkins, 2009, Kiebel et al., 2009, LeCun et al., 2015). This structure follows from generative models that entertain state transitions or sequences over time. The resulting model enables inference about narratives with deep temporal structure (c.f., sequential scene construction) of the sort seen in reading. In short, equipping an agent or simulated subject with deep temporal models allows them to accumulate evidence over different temporal scales to find the best explanation for their sensations.

**Table 1:** Applications of active inference for Markov decision processes.

Application	Comment	References
Decision making under uncertainty	Initial formulation of active inference for *Markov decision processes* and *sequential policy optimisation*	Friston et al. (2012b)
Optimal control (the mountain car problem)	Illustration of *risk sensitive or KL control* in an engineering benchmark	Friston et al. (2012a)
Evidence accumulation: Urns task	Demonstration of how beliefs states are absorbed into a generative model	FitzGerald et al., 2015b, FitzGerald et al., 2015c
Addiction	Application to psychopathology	Schwartenbeck et al. (2015c)
Dopaminergic responses	Associating dopamine with the encoding of (expected) precision provides a plausible account of dopaminergic discharges	Friston et al. (2014), FitzGerald et al. (2015a)
Computational fMRI	Using Bayes optimal precision to predict activity in dopaminergic areas	Schwartenbeck et al. (2015a)
Choice preferences and epistemics	Empirical testing of the hypothesis that people prefer to keep options open	Schwartenbeck et al. (2015b)
Behavioural economics and trust games	Examining the effects of prior beliefs about self and others	Moutoussis et al. (2014)
Foraging and two step mazes	Formulation of epistemic and pragmatic value in terms of *expected free energy*	Friston et al. (2015)
Habit learning, reversal learning and devaluation	Learning as minimising variational free energy with respect to model parameters – and action selection as *Bayesian model averaging*	FitzGerald et al. (2014), Friston et al. (2016)
Saccadic searches and scene construction	*Mean field approximation* for multifactorial hidden states, enabling high dimensional beliefs and outcomes: c.f., functional segregation	Friston and Buzsaki (2016), Mirza et al. (2016)
Electrophysiological responses: *place-cell activity, omission related responses, mismatch negativity, P300, phase-procession, theta-gamma coupling*	Simulating neuronal processing with a gradient descent on variational free energy; c.f., dynamic *Bayesian belief propagation* based on marginal free energy	In press
Structure learning, sleep and insight	Inclusion of parameters into expected free energy to enable structure learning via *Bayesian model reduction*	Under review
Narrative construction and reading	Hierarchical generalisation of generative model with *deep temporal structure*	Current paper

This paper has two agendas: to introduce hierarchical (deep) generative models for active inference under Markov decision processes (or hidden Markov models) and to show how their belief updating can be understood in terms of neuronal processes. The problem we focus on is how subjects deploy active vision to disambiguate the causes of their sensations. In other words, we ask how people choose where to look next, when resolving uncertainty about the underlying conceptual, semantic or lexical causes of sensory input. This means that we are not concerned with computational linguistics *per se* but the more general problem of *epistemic foraging*, while using reading as an example.

Epistemics is at the heart of active inference, which is all about reducing surprise or uncertainty, where uncertainty is expected surprise. Technically, this means that one can describe both inference (perception) and behaviour (action) in terms of minimising a free energy functional of probabilistic or Bayesian beliefs. In this setting, variational free energy approximates surprise and expected free energy approximates uncertainty (a.k.a. entropy). This single imperative provides an inclusive account of established (normative) approaches to perception and action; for example, the principle of maximum mutual information, the principle of minimum redundancy, formulations of saliency as Bayesian surprise, risk sensitive or KL control, expected utility theory, and so on (Barlow, 1974, Itti and Baldi, 2009, Kappen et al., 2012, Ortega and Braun, 2013). Our focus here is on how subjects use accumulated beliefs about the hidden states of the world to prescribe active sampling of new information to resolve their uncertainty quickly and efficiently (Ferro et al., 2010).

Our second agenda is to translate these normative (variational) principles into neurobiology by trying to establish the construct validity of active inference in terms of behaviour and electrophysiological responses. We do this at three levels: first, by highlighting the similarity between the message passing implied by minimising variational free energy and the neurobiology of neuronal circuits. Specifically, we try to associate the dynamics of a gradient descent on variational free energy with neuronal dynamics based upon neural mass models (Lopes da Silva, 1991). Furthermore, the exchange of sufficient statistics implicit in belief propagation is compared with the known characteristics of extrinsic (between cortical area) and intrinsic (within cortical area) neuronal connectivity. Second, we try to reproduce reading-like behaviour – in which epistemically rich information is sampled by sparse, judicious saccadic eye movements. This enables us to associate perisaccadic updating with empirical phenomena, such as delay period activity and perisaccadic local field potentials (Kojima and Goldman-Rakic, 1982, Purpura et al., 2003, Pastalkova et al., 2008). Finally, in terms of the non-invasive electrophysiology, we try to reproduce the well-known violation responses indexed by phenomena like the mismatch negativity (MMN) and P300 waveforms in event related potential research (Strauss et al., 2015).

This paper comprises four sections. The first (Active inference and free energy) briefly reviews active inference, establishing the normative principles that underlie action and perception. The second section (Belief propagation and neuronal networks) considers action and perception, paying special attention to hierarchical generative models and how the minimisation of free energy could be implemented in the brain. The third section (Simulations of reading) introduces a particular generative model used to simulate reading and provides an illustration of the ensuing behaviour – and simulated electrophysiological responses. The final section (Simulations of classical violation responses) rehearses the reading simulations using different prior beliefs to simulate responses to violations at different hierarchical levels in the model.

## 2 Active inference and free energy

Active inference rests upon a generative model that is used to infer the most likely causes of observable outcomes in terms of expected states of the world. A generative model is just a probabilistic specification of how consequences (outcomes) follow from causes (states). These states are called latent or *hidden* because they can only be inferred through observations. Clearly, observations depend upon action (e.g., where you are looking). This requires the generative model to represent outcomes under different actions or policies. Technically, expectations about (future) outcomes and their hidden causes are optimised by minimising variational free energy, which renders them the most likely (posterior) expectations about the (future) states of the world, given (past) observations. This follows because the variational free energy is an upper bound on (negative) log Bayesian model evidence; also known as surprise, surprisal or self-information (Dayan et al., 1995). Crucially, the prior probability of each policy (i.e., action or plan) is the free energy expected under that policy (Friston et al., 2015). This means that policies are more probable if they minimise expected surprise or resolve uncertainty.

Evaluating the expected free energy of plausible policies – and implicitly their posterior probabilities – enables the most likely action to the selected. This action generates a new outcome and the cycle of perception and action starts again. The resulting behaviour represents a principled sampling of sensory cues that has epistemic, uncertainty reducing and pragmatic, surprise reducing aspects. The pragmatic aspect follows from prior beliefs or preferences about future outcomes that makes some outcomes more surprising than others. For example, I would not expect to find myself dismembered or humiliated – and would therefore avoid these surprising state of affairs. On this view, behaviour is dominated by epistemic imperatives until there is no further uncertainty to resolve. At this point pragmatic (prior) preferences predominate, such that explorative behaviour gives way to exploitative behaviour. In this paper, we focus on epistemic behaviour and only use prior preferences to establish a task or instruction set. Namely, to report a categorical decision when sufficiently confident; i.e., under the prior belief one does not make mistakes.

### 2.1 Hierarchical generative models

**Fig. 1:**
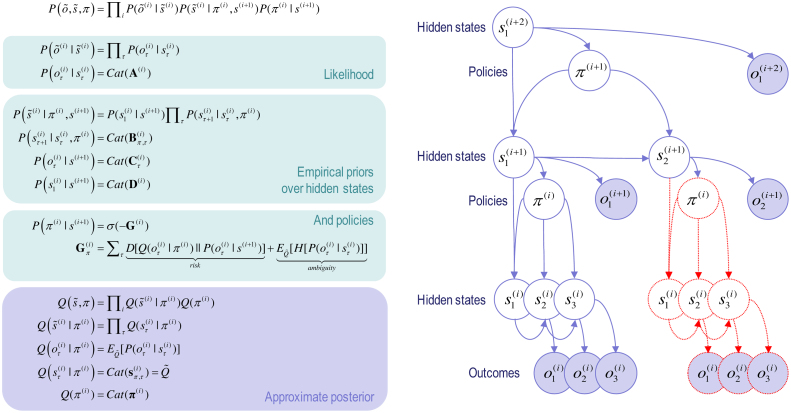
**Generative model and (approximate) posterior**. **Left panel**: these equations specify the generative model. A generative model is the joint probability of outcomes or consequences and their (latent or hidden) causes, see top equation. Usually, the model is expressed in terms of a *likelihood* (the probability of consequences given causes) and *priors* over causes. When a prior depends upon a random variable it is called an *empirical prior*. Here, the likelihood is specified by an array **A** whose elements are the probability of an outcome under every combination of hidden states. The empirical priors pertain to probabilistic transitions (in the **B** arrays) among hidden states that can depend upon action, which is determined probabilistically by policies (sequences of actions encoded by *π*). The key aspect of this generative model is that policies are more probable *a priori* if they minimise the (path integral of) expected free energy **G,** which depends upon our prior preferences about outcomes encoded by the array **C**. Finally, the **D** arrays specified the initial state, given the state of the level above. This completes the specification of the model in terms of parameter arrays that constitute **A**, **B**, **C** and **D**. Bayesian model inversion refers to the inverse mapping from consequences to causes; i.e., estimating the hidden states and other variables that cause outcomes. In variational Bayesian inversion, one has to specify the form of an approximate posterior distribution, which is provided in the lower panel. This particular form uses a mean field approximation, in which posterior beliefs are approximated by the product of marginal distributions over hierarchical levels and points in time. Subscripts index time (or policy), while (bracketed) superscripts index hierarchical level. See the main text and Table 2 for a detailed explanation of the variables (italic variables represent hidden states, while bold variables indicate expectations about those states). **Right panel**: this Bayesian graph represents the conditional dependencies among hidden states and how they cause outcomes. Open circles are random variables (hidden states and policies) while filled circles denote observable outcomes. The key aspect of this model is its hierarchical structure that represents sequences of hidden states over time or epochs. In this model, hidden states at higher levels generate the initial states for lower levels – that then unfold to generate a sequence of outcomes: c.f., associative chaining (Page and Norris, 1998). Crucially, lower levels cycle over a sequence for each transition of the level above. This is indicated by the variables outlined in red, which are ‘reused’ as higher levels unfold. It is this scheduling that endows the model with deep temporal structure. Note that hidden states at any level can generate outcomes and hidden states at the lower level. Furthermore, the policies at each level depend upon the hidden states of the level above – and are in play for the sequence of state transitions at the level below. This means that hidden states can influence subordinate states in two ways: by specifying the initial states – or via policy-dependent state transitions. Please see main text and Table 2 for a definition of the variables. For clarity, time subscripts have been omitted from hidden states at level *i* + 1. (For interpretation of the references to colour in this figure legend, the reader is referred to the web version of this article.)

We are concerned here with hierarchical generative models in which the outcomes of one level generate the hidden states at a lower level. Fig. 1 provides a schematic of this sort of model. Outcomes depend upon hidden states, while hidden states unfold in a way that depends upon a sequence of actions or a *policy.* The generative model is specified by two sets of matrices (or arrays). The first set **A**^(*i*,*m*)^, maps from hidden states to the *m*-th outcome or modality at the *i*-th level; for example, exteroceptive (e.g., visual) or proprioceptive (e.g., eye position) observations. The second set: **B**^(*i*,*n*)^(*u*), prescribes the transitions among the *n*-th hidden state or factor, at the *i*-th level, under action *u*. Hidden factors correspond to different states of the world, such as the location (i.e., where) and category (i.e., what) of an object. Hierarchical levels are linked by **D**^(*i*,*n*)^ that play a similar role to **A**^(*i*,*m*)^. However, instead of mapping from hidden states to outcomes they map from hidden states at the given level to the *initial states* of the *n*-th factor at the level below. A more detailed description of these parameters can be found in Table 2 and the Appendix A. For simplicity, Fig. 1 assumes there is a single hidden factor and outcome modality.

**Table 2:** Glossary of expressions (for the *i*-th hierarchical level of a generative model).

Expression	Description
	Outcomes in *M* modalities at each time point, taken to be ‘one-in-K’ vectors of dimension *D*(*i*,*m*)
	Sequences of outcomes until the current time point
	Hidden states of the *n*-th factor at each time point and their posterior expectations under each policy
	Sequences of hidden states until the end of the current sequence
	Sequential policies specifying controlled transitions within *N* hidden factors over time and their posterior expectations
	Action or control variables for the *n*-th factor of hidden states at a particular time specified by a policy
	Auxiliary (depolarisation) variable corresponding to the surprise of an expected state – a softmax function of depolarisation
	Predictive posterior over future outcomes using a generalised dot product (sum of products) operator
	Bayesian model average of hidden states over policies
	Likelihood tensor mapping from hidden states to the *m*-th modality
	Transition probability for the *n*-th hidden state under an action (prescribed by a policy at a particular time)
	Prior probability of the *m*-th outcome at the *i*-th level conditioned on the *n*-th (hierarchical) context
	Prior probability of the *n*-th initial state at the *i*-th level conditioned on the *n*-th (hierarchical) context
	Marginal free energy for each policy
	Expected free energy for each policy
	Entropy of outcomes under each combination of states in the *m*-th modality

The generative model in Fig. 1 generates outcomes in the following way: first, a policy (action or plan) is selected at the highest level using a softmax function of their expected free energies. Sequences of hidden states are then generated using the probability transitions specified by the selected policy (encoded in **B** matrices). These hidden states generate outcomes and initial hidden states in the level below (according to **A** and **D** matrices). In addition, hidden states can influence the expected free energy (through **C** matrices) and therefore influence the policies that determine transitions among subordinate states. The key aspect of this generative model is that state transitions proceed at different rates at different levels of the hierarchy. In other words, the hidden state at a particular level entails a sequence of hidden states at the level below. This is a necessary consequence of conditioning the initial state at any level on the hidden states in the level above. Heuristically, this hierarchical model generates outcomes over nested timescales; like the second-hand of a clock that completes a cycle for every tick of the minute-hand that, in turn precesses more quickly than the hour hand. It is this particular construction that lends the generative model a deep temporal architecture. In other words, hidden states at higher levels contextualise transitions or trajectories of hidden states at lower levels; generating a deep dynamic narrative.

### 2.2 Variational free energy and inference

For any given generative model, active inference corresponds to optimising expectations of hidden states and policies with respect to variational free energy. These expectations constitute the sufficient statistics of posterior beliefs, usually denoted by the probability distribution , where  are hidden or unknown states and policies. This optimisation can be expressed mathematically as:(1)
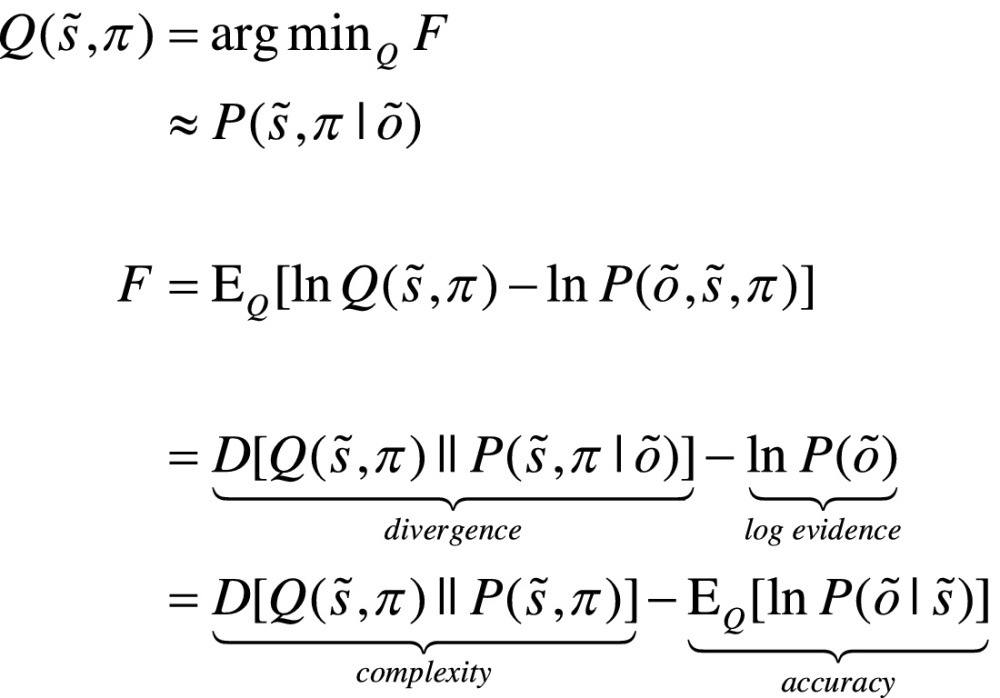
where  denotes observations up until the current time point and  represents hidden states over all the time points in a sequence. Because the (KL) divergence between a subject’s beliefs and the true posterior cannot be less than zero, the penultimate equality means that free energy is minimised when the two are the same. At this point, the free energy becomes the *surprise* or negative log evidence for the generative model (Beal, 2003). In other words, minimising free energy is equivalent to minimising the complexity of accurate explanations for observed outcomes.

In active inference, both beliefs and action minimise free energy. However, beliefs cannot affect outcomes. This means that action affords the only means of minimising surprise, where action minimises expected free energy; i.e. expected surprise or uncertainty. In turn, this rests on equipping subjects with the prior beliefs that their policies will minimise expected free energy (Friston et al., 2015):(2)
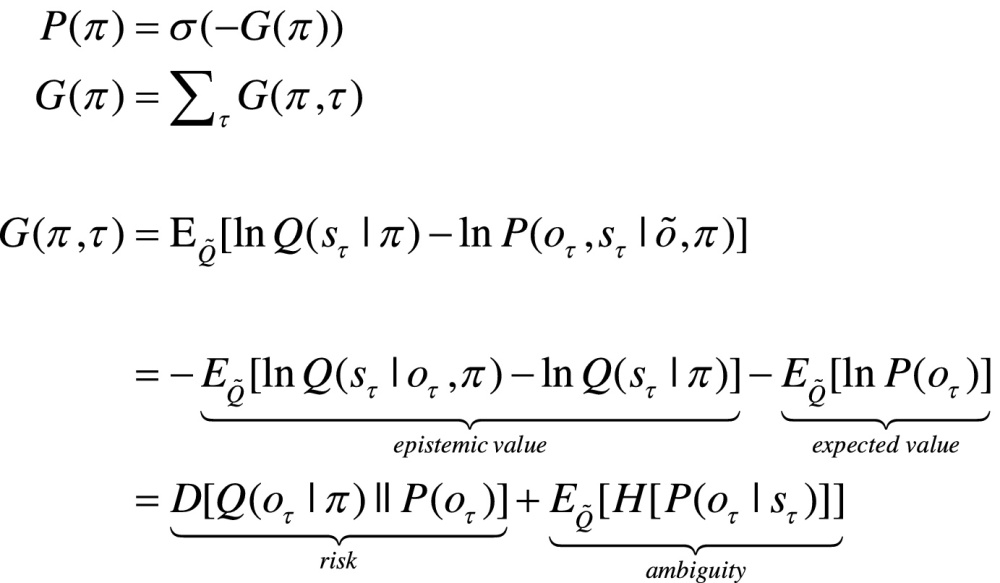
Here, *G*(*π*,*τ*) denotes the expected free energy of a particular policy at a particular time, and  is the predictive distribution over hidden states and outcomes under that policy. Comparing the expressions for expected free energy (Eq. (2)) with variational free energy (Eq. (1)), we see that the (negative) divergence becomes *epistemic value* and the *log evidence* becomes *expected value* – provided we associate the prior preference over future outcomes with value. In other words, valuable outcomes are those we expect to encounter and costly outcomes are surprising (e.g., being in pain). The last equality provides a complementary interpretation; in which complexity becomes *risk*, and inaccuracy becomes *ambiguity*. Please see the appendices for derivations.

There are several special cases of expected free energy that appeal to (and contextualise) established constructs. For example, maximising epistemic value is equivalent to maximising (expected) Bayesian surprise (Itti and Baldi, 2009), where Bayesian surprise is the divergence between posterior and prior beliefs. This can also be interpreted as the principle of maximum mutual information or minimum redundancy (Barlow, 1961, Linsker, 1990, Olshausen and Field, 1996, Laughlin, 2001). In this context, epistemic value is the expected *mutual information* between future states and their consequences, which is also known as *information gain*. Because epistemic value (i.e., mutual information) cannot be less than zero, it disappears when the (predictive) posterior ceases to be informed by new observations. This means epistemic behaviour will search out observations that resolve uncertainty (e.g., foraging to find a prey or turning on the light in a dark room). However, when the agent is confident about the state of the world, there can be no further information gain and pragmatic (prior) preferences dominate. Crucially, epistemic and expected values have a definitive quantitative relationship, which means there is no need to adjudicate between explorative, epistemic uncertainty reducing and exploitative, pragmatic goal directed behaviour. The switch between behavioural policies emerges naturally from minimising expected free energy. This switch depends on the relative contribution of epistemic and expected value, thereby resolving the exploration-exploitation dilemma. Furthermore, in the absence of any precise preferences, purposeful behaviour is purely epistemic in nature. In what follows, we will see that prior preferences or goals are usually restricted to the highest levels of a hierarchy. This means that active inference at lower levels is purely uncertainty reducing, where action ceases when uncertainty approaches zero (in this paper, a sequence of actions terminates when the uncertainty about states prescribed by higher levels is  nats or less).

### 2.3 Summary

Minimising expected free energy is essentially the same as avoiding surprises and resolving uncertainty. This resolution of uncertainty is closely related to satisfying artificial curiosity (Schmidhuber, 1991, Still and Precup, 2012) and speaks to the value of information (Howard, 1966). Expected free energy can be expressed in terms of epistemic and expected value – or in terms of risk and ambiguity. The expected complexity or risk is exactly the same quantity minimised in risk sensitive or KL control (Klyubin et al., 2005, van den Broek et al., 2010), and underpins related (free energy) formulations of bounded rationality based on complexity costs (Braun et al., 2011, Ortega and Braun, 2013). In other words, minimising expected complexity renders behaviour risk-sensitive, while maximising expected accuracy induces ambiguity-resolving behaviour. In the next section, we look more closely at how this minimisation is implemented.

## 3 Belief propagation and neuronal networks

**Fig. 2:**
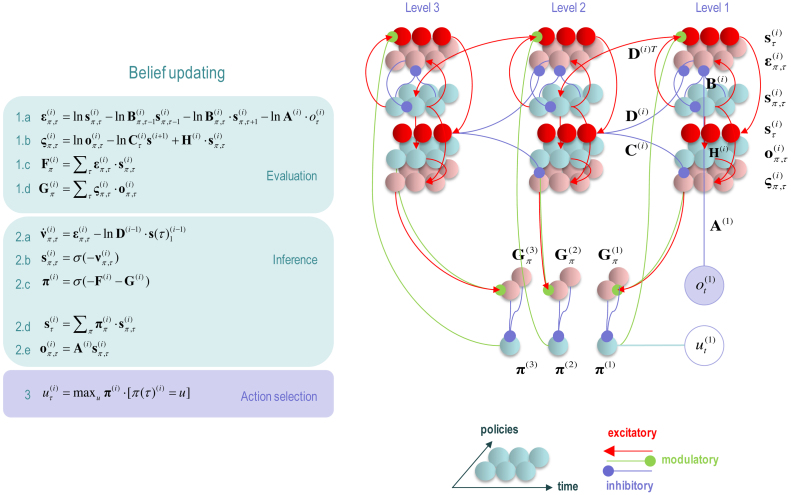
**Schematic overview of belief propagation**: **left panel**: these equalities are the belief updates mediating inference (i.e. state estimation) and action selection. These expressions follow in a fairly straightforward way from a gradient descent on variational free energy. The equations have been expressed in terms of prediction errors that come in two flavours. The first, *state* prediction error scores the difference between the (log) expected states under any policy and time (at each hierarchical level) and the corresponding predictions based upon outcomes and the (preceding and subsequent) hidden states (1.a). These represent likelihood and empirical prior terms respectively. The prediction error drives log-expectations (2.a), where the expectation *per se* is obtained via a softmax operator (2.b). The second, *outcome* prediction error reports the difference between the (log) expected outcome and that predicted under prior preferences set by the level above (plus an ambiguity term – see appendix) (1.b). This prediction error is weighted by the expected outcomes to evaluate the expected free energy (1.d). Similarly, the free energy *per se* is the expected state prediction error, under current beliefs about hidden states (1.c). These policy-specific free energies are combined to give the policy expectations via a softmax function (2.c). Finally, expectations about hidden states are a Bayesian model average over expected policies (2.d) and expectations about policies specify the action that is most likely to realise the expected outcome (3). The (Iverson) brackets in Eq. (3) return one if the condition in square brackets is satisfied and zero otherwise. **Right panel**: this schematic represents the message passing implicit in the equations on the left. The expectations have been associated with neuronal populations (coloured balls) that are arranged to highlight the correspondence with known intrinsic (within cortical area) and extrinsic (between cortical areas) connections. Red connections are excitatory, blue connections are inhibitory and green connections are modulatory (i.e., involve a multiplication or weighting). This schematic illustrates three hierarchical levels (which are arranged horizontally in this figure, as opposed to vertically in Fig. 1), where each level provides top-down empirical priors for the initial state of the level below, while the lower level supplies evidence for the current state at the level above. The intrinsic connections mediate the empirical priors and Bayesian model averaging. Cyan units correspond to expectations about hidden states and (future) outcomes under each policy, while red states indicate their Bayesian model averages. Pink units correspond to (state and outcome) prediction errors that are averaged to evaluate (variational and expected) free energy and subsequent policy expectations (in the lower part of the network). This (neuronal) network interpretation of belief updating means that connection strengths correspond to the parameters of the generative model in Fig. 1. Please see Table 2 for a definition of the variables. The variational free energy has been omitted from this figure because the policies in this paper differ only in the next action. This means the evidence (i.e. variational free energy) from past outcomes is the same for all policies. (For interpretation of the references to colour in this figure legend, the reader is referred to the web version of this article.)

Having defined a generative model, the expectations encoding posterior beliefs (and action) can be optimised by minimising variational free energy. Fig. 2 provides the mathematical expressions for this optimisation or belief updating. Although the updates look a little complicated, they are remarkably plausible in terms of neurobiological process theories (Friston et al., 2014). In brief, minimising variational free energy means that expectations about allowable policies become a softmax function of variational and expected free energy, where the (path integral) of variational free energy scores the evidence that a particular policy is being pursued (Eq. 1.c in Fig. 2). Conversely, the expected free energy plays the role of a prior over policies that reflect their ability to resolve uncertainty (Eq. 1.d). The resulting policy expectations are used to predict the state at each level in the form of a *Bayesian model average*; in other words, the expected states under each policy are combined in proportion to the expected probability of each policy (Eq. 2.d). These Bayesian model averages then provide (top-down) prior constraints on the initial states of the level below. Finally, expectations about policies enable the most likely action to be selected at each level of the hierarchy. Fig. 2 only shows action selection for the lowest (first) level.

Of special interest here, are the updates for expectations of hidden states (for each policy and time). These have been formulated as a gradient descent on variational free energy (see Appendix A1). This furnishes a dynamical process theory that can be tested against empirical measures of neuronal dynamics. Specifically, the Bayesian updating or belief propagation (see Appendix A2) has been expressed so that it can be understood in terms of neurophysiology. Under this interpretation, expected states are a softmax function of log expectations that can be associated with neuronal depolarisation (Eq. 2.b). In other words, the softmax function becomes a firing rate function of depolarisation, where changes in postsynaptic potential are caused by currents induced by presynaptic input from prediction error units (Eq. 2.a). In this formulation, *state* prediction errors are the difference between the log expected state and its prediction from observed outcomes, the preceding state and subsequent state (Eq. 1.a). Similarly, *outcome* prediction errors are the difference between the log expected outcome and the outcome predicted by hidden states in the level above (Eq. 1.b). Physiologically, this means that when state prediction error unit activity is suppressed, there is no further depolarisation of expectation units and their firing attains a variational free energy minimum. This suggests that for every expectation unit there should be a companion error unit, whose activity is the rate of change of depolarisation of the expectation unit; for example, excitatory (expectation) pyramidal cells and fast spiking inhibitory (error) interneurons (Sohal et al., 2009, Cruikshank et al., 2012, Lee et al., 2013).

### 3.1 Extrinsic and intrinsic connectivity

**Fig. 3:**
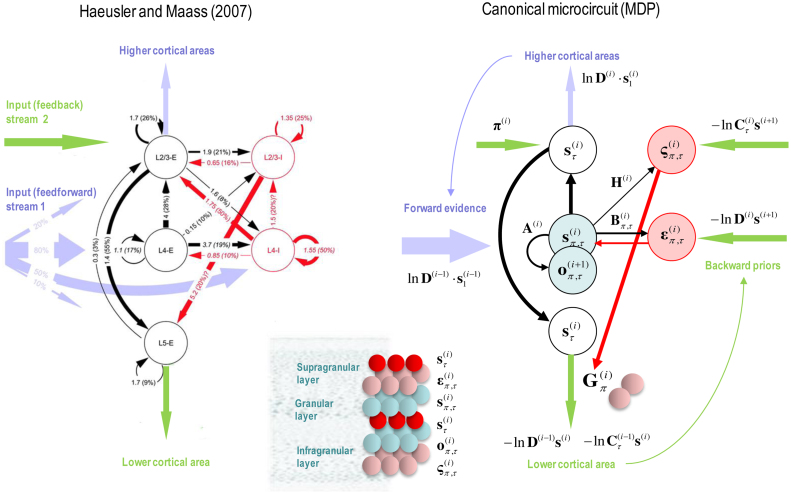
**Belief propagation and intrinsic connectivity**. This schematic features the correspondence between known canonical microcircuitry and the belief updates in Fig. 2. **Left Panel**: a canonical microcircuit based on (Haeusler and Maass, 2007), where inhibitory cells have been omitted from the deep layers – because they have little interlaminar connectivity. The numbers denote connection strengths (mean amplitude of PSPs measured at soma in mV) and connection probabilities (in parentheses) according to (Thomson and Bannister, 2003). **Right panel**: the equivalent microcircuitry based upon the message passing scheme of the previous figure. Here, we have placed the outcome prediction errors in superficial layers to accommodate the strong descending (inhibitory) connections from superficial to deep layers. This presupposes that descending (interlaminar) projections disinhibit Layer 5 pyramidal cells that project to the medium spiny cells of the striatum (Arikuni and Kubota, 1986). The computational assignments in this figure should be compared with the equivalent scheme for predictive coding in (Bastos et al., 2012). The key difference is that superficial excitatory (e.g., pyramidal) cells encode expectations of hidden states, as opposed to state prediction errors. This is because the prediction error is encoded by their postsynaptic currents, as opposed to their depolarisation or firing rates (see main text). The white circles correspond to the Bayesian model average of state expectations, which are the red balls in the previous figures (and the inset). Black arrows denote excitatory *intrinsic* connections, while red arrows are inhibitory. Blue arrows denote bottom-up of ascending *extrinsic* connections, while green arrows are top-down or descending. (For interpretation of the references to colour in this figure legend, the reader is referred to the web version of this article.)

The graphics in Fig. 2 have assigned various expectations and errors to neuronal populations in specific cortical layers. This (speculative) assignment, allows one to talk about the functional anatomy of extrinsic and intrinsic connectivity in terms of belief propagation. In brief, the mathematical form of Bayesian belief updating tells us which neuronal representations talk to each other. For example, in a hierarchical setting, the only sufficient statistics that are exchanged between levels are the Bayesian model averages of expected states. This means, by definition, that the Bayesian model averages must be encoded by principal cells that send neuronal connections to other areas in the cortical hierarchy. These are the superficial and deep pyramidal cells show in red in Fig. 2. Next, we know that the targets of ascending extrinsic (feedforward) connections from superficial pyramidal cells are the spiny stellate cells in Layer 4 (Felleman and Van Essen, 1991, Bastos et al., 2012, Markov et al., 2013). The only sufficient statistics in receipt of Bayesian model averages from the level below are policy-specific expectations about hidden states. These can be associated with spiny stellate cells (upper cyan layer in Fig. 2). These sufficient statistics are combined to form the Bayesian model average in superficial pyramidal cells, exactly as predicted by quantitative connectivity studies of the canonical cortical microcircuit (Thomson and Bannister, 2003). One can pursue this game and – with some poetic license – reproduce the known quantitative microcircuitry of inter-and intralaminar connections. Fig. 3 illustrates one solution that reproduces not only the major intrinsic connections but also their excitatory and inhibitory nature. This arrangement suggests that inhibitory interneurons play the role of error units (which is consistent with the analysis above), while policy-specific expectations are again encoded by excitatory neurons in Layer 4. Crucially, this requires a modulatory weighting of the intrinsic feedforward connections from expectation units to their Bayesian model averages in the superficial layers. This brings us to extrinsic connections and the neuronal encoding of policies in the cortico-basal ganglia-thalamic loops.

### 3.2 Extrinsic connectivity and cortico-subcortical loops

According to the belief propagation equations, expected policies rest upon their variational and expected free energy. These free energies comprise (KL) divergences that can always be expressed in terms of an average prediction error. Here, the variational free energy is the expected state prediction error, while the expected free energy is the expected outcome prediction error. These averages are gathered over all policies and time points within a hierarchical level and are passed through a sigmoid (softmax) function to produce policy expectations. If we associate this pooling with cortico-subcortical projections to the basal ganglia – and the subsequent Bayesian model averaging with thalamocortical projections to the cortex – there is a remarkable correspondence between the implicit connectivity (both in terms of its specificity and excitatory versus inhibitory nature) and the connectivity of the cortico-basal ganglia-thalamocortical loops.

**Fig. 4:**
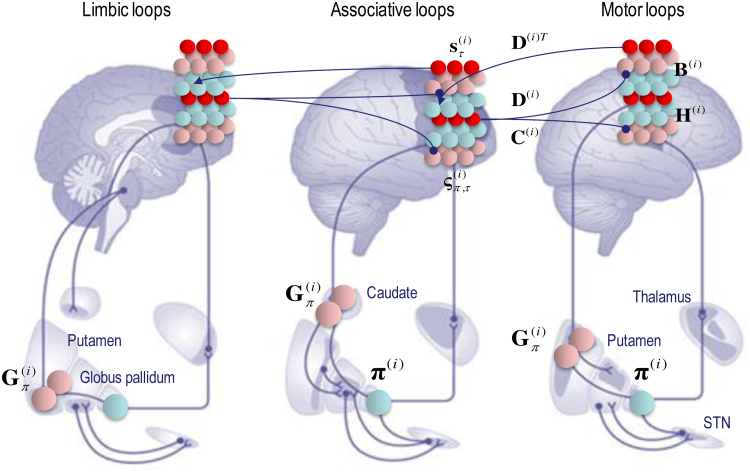
**Belief propagation and extrinsic connectivity**. This schematic illustrates a putative mapping between expectations that are updated during belief updating and recurrent interactions within the cortico-basal ganglia-thalamic loops. This figure is based upon the functional neuroanatomy described in (Jahanshahi et al., 2015), which assigns motor updates to motor and premotor cortex projecting to the putamen; associative loops to prefrontal cortical projections to the caudate and limbic loops to projections to the ventral striatum. The correspondence between the message passing implicit in belief propagation and the organisation of these loops is remarkable; even down to the level of the sign (excitatory or inhibitory) of the neuronal connections. The striatum (caudate and putamen) and the subthalamic nucleus (STN) receive inputs from many cortical and subcortical areas. The internal segment of the globus pallidus (GPi) constitutes the main output nucleus from the basal ganglia. The basal ganglia are not only connected to motor areas (motor cortex, supplementary motor cortex, premotor cortex, cingulate motor area and frontal eye fields) but also have connections with associative cortical areas. The basal ganglia nuclei have topologically organized motor, associative and limbic territories; the posterior putamen is engaged in sensorimotor function, while the anterior putamen (or caudate) and the ventral striatum are involved in associative (cognitive) and limbic (motivation and emotion) functions (Jahanshahi et al., 2015).

The schematic in Fig. 4 is based upon the hierarchical anatomy of cortico-basal ganglia-thalamic loops described in (Jahanshahi et al., 2015). If one subscribes to this functional anatomy, the formal message passing of belief propagation suggests that competing low level (motor executive) policies are evaluated in the putamen; intermediate (associative) policies in the caudate and high level (limbic) policies in the ventral striatum. These representations then send (inhibitory or GABAergic) projections to the globus pallidus interna (GPi) that encodes the expected (selected) policy. These expectations are then communicated via thalamocortical projections to superficial layers encoding Bayesian model averages. From a neurophysiological perspective, the best candidate for the implicit averaging would be matrix thalamocortical circuits that “appear to be specialized for robust transmission over relatively extended periods, consistent with the sort of persistent activation observed during working memory and potentially applicable to state-dependent regulation of excitability” (Cruikshank et al., 2012). This deep temporal hierarchy is apparent in hierarchically structured cortical dynamics – invasive recordings in primates suggest an anteroposterior gradient of spontaneous fluctuation time constants consistent with the architecture in Fig. 4 (Kiebel et al., 2008, Murray et al., 2014). Clearly, there are many anatomical issues that have been ignored here; such as the distinction between direct and indirect pathways (Frank, 2005), the role of dopamine in modulating the precision of beliefs about policies (Friston et al., 2014) and so on. However, the basic architecture suggested by the above treatment speaks to the biological plausibility of belief updating under hierarchical generative models.

### 3.3 Summary

By assuming a generic (hierarchical Markovian) form for the generative model, it is fairly easy to derive Bayesian updates that clarify the relationship between perception and action selection. In brief, the agent first infers the hidden states under each policy that it entertains. It then evaluates the evidence for each policy based upon observed outcomes and beliefs about future states. The posterior beliefs about each policy are used to form a Bayesian model average of the next outcome, which is realised through action. In hierarchical models, the implicit belief updating (known as belief propagation in machine learning) appears to rest on message passing that bears a remarkable similarity to cortical hierarchies and cortico-basal ganglia-thalamic loops; both in terms of extrinsic connectivity and intrinsic canonical (cortical) microcircuits. In the next section, we use this scheme to simulate reading.

## 4 Simulations of reading

**Fig. 5:**
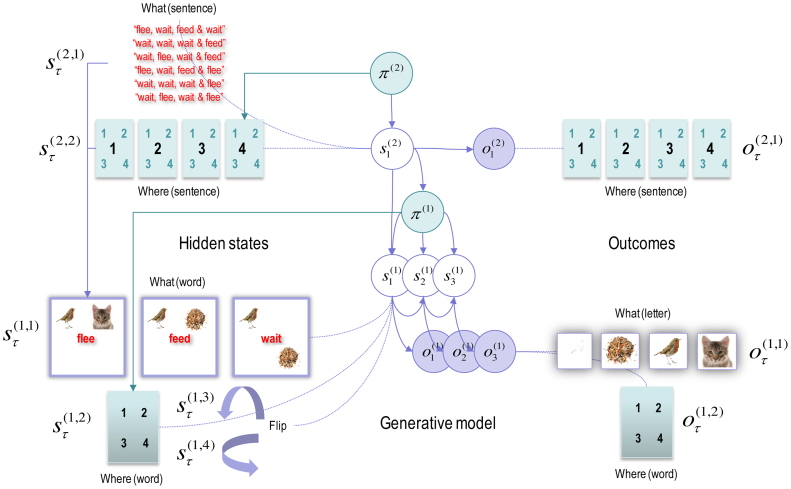
**The generative model used to simulate reading**. This graphical model shows the conditional dependencies of the generative model used in subsequent figures, using the same format as Fig. 1. In this model there are two hierarchical levels with three hidden states at the second level and four at the first level (hidden states and outcomes pertaining to categorical decisions and feedback have been omitted for clarity). The hidden states at the higher level correspond to the sentence or narrative – generating sequences of *words* at the first level – and which *word* the agent is currently sampling (with six alternative *sentences* and four *words* respectively). These (higher level) hidden states combine to specify the word generated at the first level (*flee*, *feed* or *wait*). The hidden states at the first level comprise the current *word* and which quadrant the agent is looking at. These hidden states combine to generate outcomes in terms of *letters* or icons that would be sampled if the agent looked at a particular *location* in the current *word.* In addition, two further hidden states provide a local feature context by flipping the locations vertically or horizontally. The vertical flip can be thought of in terms of font substitution (upper case versus lowercase), while the horizontal flip means a word is invariant under changes to the order of the letters (c.f., palindromes that read the same backwards as forwards). In this example, *flee* means that a bird is next to a cat, *feed* means a bird is next to some seeds and *wait* means seeds are above (or below) the bird. Notice that there are outcomes at both levels. At the higher level there is a (proprioceptive) outcome signalling the *word* currently being sampled (e.g., head position), while at the lower level there are two outcome modalities. The first (exteroceptive) outcome corresponds to the observed letter and the second (proprioceptive) outcome reports the letter location (e.g., direction of gaze in a head-centred frame of reference). Similarly, there are policies at both levels. The high-level policy determines which word the agent is currently reading, while the lower level dictates the transitions among the quadrants containing letters.

The remainder of this paper considers simulations of reading using a generative model that is a hierarchical extension of a model we have used previously to illustrate scene construction (Mirza et al., 2016). In the original paradigm, (simulated) subjects had to sample four quadrants of a visual scene to classify the arrangement of visual objects (a *bird*, a *cat* and *seeds*) into one of three categories (*flee*, *feed* or *wait*). If the *bird* and *cat* were next to each other (in the upper or lower quadrants) the category was *flee*. If the *bird* was next to the *seeds*, the category was *feed*. Alternatively, if the *bird* and *seeds* occupied diagonal quadrants, the category was *wait*. Here, we treat the visual objects as letters and the scene as a word; enabling us to add a hierarchical level to generate sentences or sequences of words. The subject’s task was to categorise sentences of four words into *happy* or *sad* narratives; where *happy* narratives concluded with a *feed* or *wait* in the final two words. Somewhat arbitrarily, we restricted the hypotheses at the second level to 6 sentences (see Fig. 5). By stimulating reading, we hoped to produce realistic sequences of saccadic eye movements, in which the subject interrogated local features (i.e. letters) with sparse and informative foveal sampling; in other words, jumping to key letter features and moving to the next word as soon as the current word could be inferred confidently. Furthermore, because the subject has a deep model, she already has in mind the words and letters that are likely to be sampled in the future; enabling an efficient foraging for information.

To simulate this sort of task, one needs to specify the hidden factors, allowable policies and prior preferences. Fig. 5 illustrates the factorisation and hierarchical structure of the resulting model. At the highest level there are three hidden factors (only two are shown in the figure for simplicity). These comprise the sentence (with six alternatives), the word the subject is currently examining (one of four words) and the decision (*undecided*, *happy* or *sad*). The word location and current sentence specify the hidden state (word) at the lower hierarchical level. The lower level also includes a letter location state (one of four quadrants) and two spatial transformations (horizontal and vertical *flip*). The current word and letter location specify the outcome (letter or visual object; *cat*, *bird*, *seed* or *nothing*). At both the higher (e.g., sentence) and lower (e.g., word) levels, the hidden locations also specify a proprioceptive outcome in terms of higher (e.g., head) and lower (e.g., eye) movements that sample the word and letter respectively. Finally, the hidden decision state determines (e.g. auditory) feedback with three possibilities; namely, *nothing*, *right* or *wrong*. The decision state and feedback outcomes have been omitted from Fig. 5 for clarity.

This setup defines the state space and mapping from hidden states to outcomes encoded by the **A** parameters. Note that the likelihood mapping involves interactions among hidden states; for example, one has to know both the location being sampled and the word generating outcomes before the letter is specified. These interactions are modelled very simply by placing a one at the appropriate combination of hidden states (and zeros elsewhere) in the row of **A** corresponding to the outcome. Similarly, the **D** parameters specify the outcome in terms of hidden states at the lower level in terms of (combinations of) hidden factors at the higher level.

It is now necessary to specify the contingencies and transitions among hidden states in terms of the **B** parameters. There is a separate **B** matrix for every hidden factor and policy. In this example, these matrices have a very simple form: on any given trial, policies cannot change the sentence or word. This means the corresponding **B** matrices are identity matrices. For hidden locations, the **B** matrices simply encode a transition from the current location to the location specified by the policy. Here the policies were again very simple; namely, where one looks next (in a body and head centred frame of reference at the first and second levels respectively). For simplicity, the preceding actions that constitute each policy were the actions actually selected. In more sophisticated setups, policies can include different sequences of actions; however here, the number of policies and actions were the same. This means we do not have to worry about the evidence the different policies encoded by the variational free energy (as in the right panel of Fig. 2). There were three policies or actions at the second level; proceed to the next word or stop reading and make a categorical decision of *happy* or *sad*; resulting in *right* or *wrong* feedback.

Finally, the prior preferences encoded in the **C** parameters rendered all outcomes equally preferred, with the exception of being wrong, which was set at . In other words, the subject thought they were exp(4) ≈ 54 times less likely to be wrong than undecided or right. This aversion to making mistakes ensures the subject does not solicit feedback to resolve uncertainty about the category of the sentence. In other words, the subject has to be relatively confident – after epistemic foraging – about the underlying narrative before confirming any inference with feedback. Prior beliefs about first level hidden states, encoded in the **D** parameters, told the subject they would start at the first quadrant of the first word, with an equal probability of all sentences. Because all six sentences began with either *flee* or *wait*, the prior probability over words was implicitly restricted to *flee* or *wait*, with equal probabilities of horizontal flipping (because these priors do not depend on the higher level). The horizontal flipping corresponds to a spatial transformation, under which the meaning of the word is invariant, much like a palindrome. Conversely, the subject had a strong prior belief that there was no vertical flipping. This (low-level feature) transformation can be regarded as presenting words in upper or lower case. The prior over vertical flipping will become important later, when we switch prior beliefs to make uppercase (vertically flipped) stimuli the prior default to introduce violations of (feature) expectations.

A heuristic motivation for including hidden factors like horizontal flipping appeals to the way that we factorise hidden causes of stimuli; in other words, carve nature at its joints. The fact that we are capable of:

“raeding wrods with jubmled lettres” (Rayner et al., 2006),

suggests that horizontal flipping can be represented in a way that is conditionally independent of grapheme content.

This completes our specification of the generative model. To simulate reading, the equations in Fig. 2 were integrated using 16 iterations for each time point at each level. At the lowest level, an iteration is assumed to take 16 ms, so that each epoch or transition is about 256 ms.^1^ This is the approximate frequency of saccadic eye movements (and indeed phonemic processing in auditory language processing), meaning that the simulations covered a few seconds of simulated time. The scheduling of updates in hierarchical models presents an interesting issue. In principle, we could implement the belief updating synchronously; enabling second level expectations to be informed by first level expectations as they accumulate evidence. Alternatively, we could wait until the first level convergences and update higher levels asynchronously – so that the high-level waits until the lower level sequence completes before updating and providing (empirical) prior constraints for the initial state at the lower level. We elected to illustrate the latter (asynchronous) updating, noting that alternative (synchronous) schemes could be implemented and compared to empirical neuronal responses. Asynchronous scheduling has the advantage of computational simplicity, because it means each level can be integrated or solved by the same routine (here, **spm_MDP_VB_X.m**). This means that the sequence of posterior expectations following convergence at one level can be passed as (probabilistic) outcomes to the next, while the outcomes from the highest level enter as prior constraints on the initial states of the level below. Furthermore, we will see below that the ensuing updates bear a marked similarity to empirical (neurophysiological) responses.

**Fig. 6:**
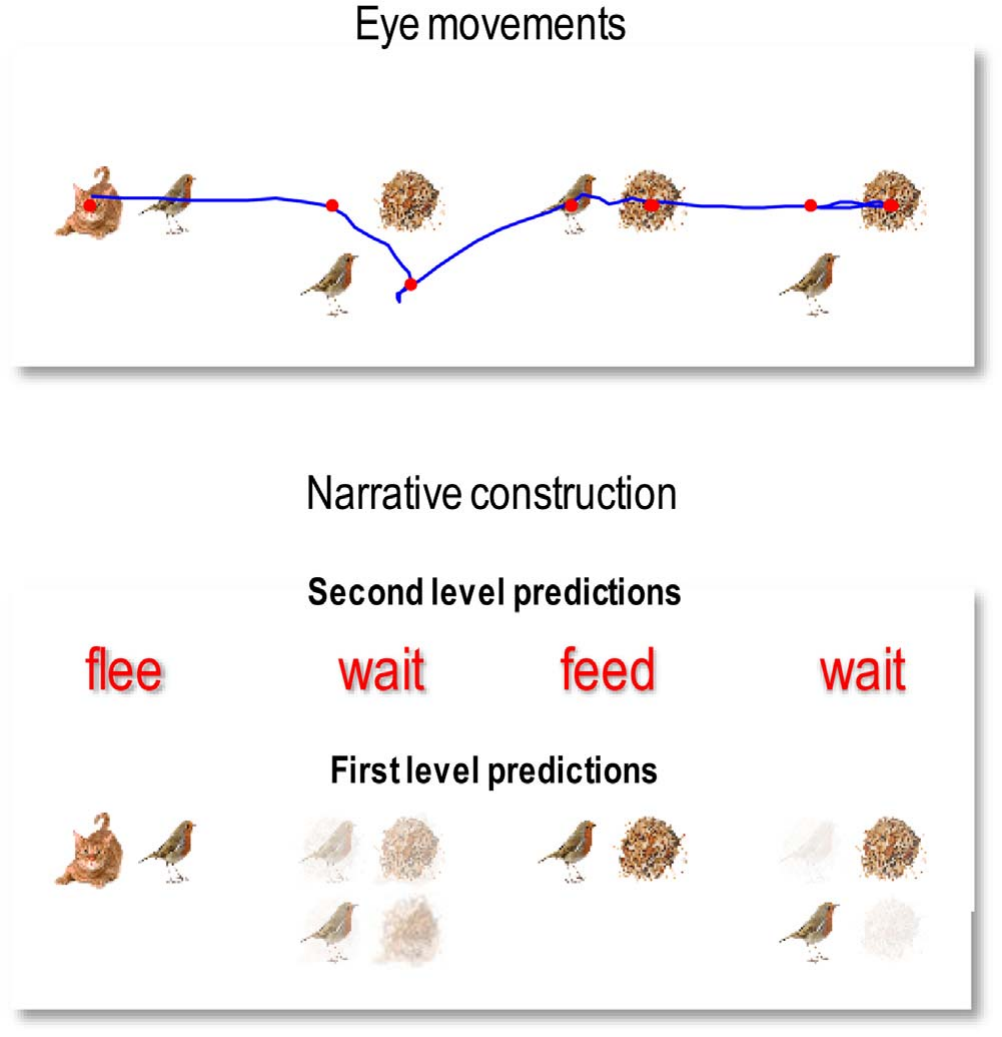
**Simulated behavioural responses during reading: upper panel**. This shows the trajectory of eye movements over four transitions at the second level that entail one or two saccadic eye movements at the first. In this trial, the subject looks at the first quadrant of the first word and sees a cat. She therefore knows immediately that the first word is *flee*. She then turns to the first quadrant of the second word and sees nothing. To resolve uncertainty she then looks at the fourth quadrant and again finds nothing, which means this word must be *wait* (because the second word of each sentence is either *flee* or *wait* – and the current word cannot be *flee* because the cat cannot be next to the bird). The next two saccades, on the subsequent word confirm the word *feed* (with the *seed* next to the *bird* sampled on the first saccade). Finally, the subject turns to the final word and discloses *seeds* after the second saccade. At this point, uncertainty about the sentence (sentence one versus sentence four) is resolved and the subject makes a correct categorisation – a *happy* story. **Lower panel**: this panel shows expected outcomes at the end of sampling each word. The upper row shows the final beliefs about the words under (correct) expectations about the sentence at the second level. This (first) sentence was “*flee, wait, feed and wait*”. At the first level, expectations about the letters under posterior beliefs about the words are shown in terms of mixtures of icons.

Fig. 6 shows simulated behavioural responses during reading in terms of eye movements (upper panel) over four transitions at the second level, where each transition entails one or two saccades at the first. In this exemplar simulation, the stimuli were generated at random using the above generative model. Here, the subject read the first sentence in lower case, apart from the second letter that was in upper case (i.e. with a surprising vertical flipping). In this trial, the subject looks at the first quadrant of the first word and sees a *cat.* She therefore knows immediately that the first word is *flee*. She then turns to the second word and sees nothing. To resolve uncertainty, she samples the fourth quadrant and again finds nothing, which means this word must be *wait* (because the second word of each sentence is either *flee* or *wait* – and the current word cannot be flee because the *cat* cannot be next to the *bird*). The next two saccades, on the subsequent word, confirm the word *feed* (with the *seed* next to the *seed* sampled on the first saccade). Finally, the subject turns to the final word and discovers *seeds* on the second saccade. At this point, residual uncertainty about the sentence is resolved and the subject makes a correct categorisation – a *happy* story. The lower panel shows expected outcomes at the end of sampling each word. The upper row shows the final beliefs about the words under (correct) expectations about the sentence of the second level. This (first) sentence was *“flee, wait*, *feed* and *wait*”.

The key thing to take from these results is that the agent can have precise beliefs about letters without ever seeing them. For example, the subject believes there is a *bird* in the second quadrant of the first word, despite the fact she never looked there. This illustrates the fact that it is not necessary to sample all the constituent letters to identify a word. Conversely, there can be uncertainty about particular letters, even though the subject is confident about the word. This is illustrated by the expectations about the letters in the second word. These expectations are consistent with *wait* but reflect a degree of uncertainty about the vertical flip (i.e., lower case or upper case font). This uncertainty – and resulting hesitancy in moving to the next word – reflects the subject’s prior belief that letters are usually presented in lower case. However, the actual stimuli were presented in a surprising way (with a vertical flip) that causes the subject to spend an extra saccade on this word, before moving to the next.

**Fig. 7:**
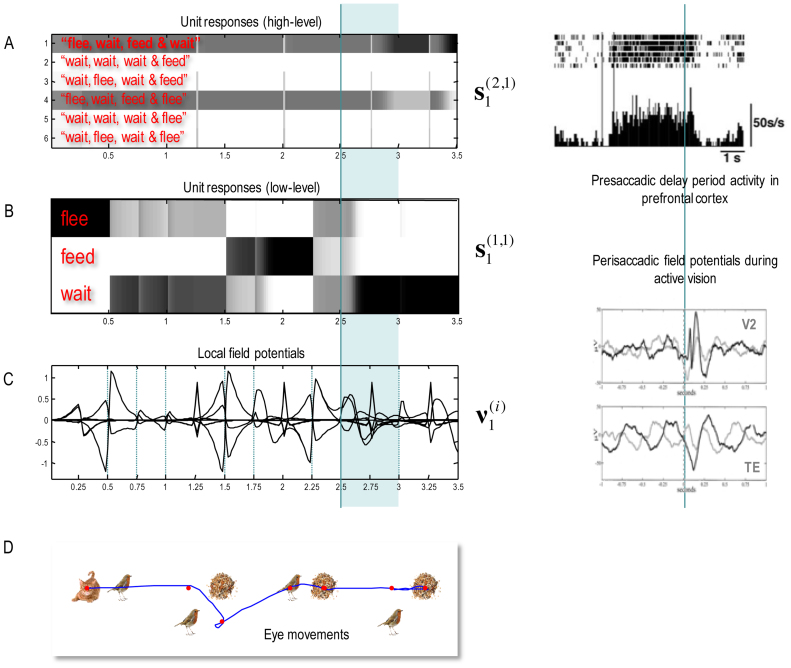
**Simulated electrophysiological responses during reading**: these panels show the Bayesian belief updating that underlies the behaviour and expectations reported in the previous figure. Expectations about the initial hidden state (at the first time step) at the higher (upper panel A) and lower (middle panel B) hierarchical levels are presented in raster format, where an expectation of one corresponds to black (i.e., the firing rate activity corresponds to image intensity). The horizontal axis is time over the entire trial, where each iteration corresponds roughly to 16 ms. The vertical axis corresponds to the six sentences at the higher level and the three words at the lower level. The resulting patterns of firing rate over time show a marked resemblance to delay period activity in the prefrontal cortex prior to saccades. Saccade onsets are shown with the vertical (cyan) lines. The inset on the upper right is based upon the empirical results reported in (Funahashi, 2014). The transients in the lower panel (C) are the firing rates in the upper panels filtered between 4 Hz and 32 Hz – and can be regarded as (band pass filtered) fluctuations in depolarisation. These simulated local field potentials are again remarkably similar to empirical responses. The examples shown in the inset are based on the study of perisaccadic electrophysiological responses during activation reported in (Purpura et al., 2003). The upper traces come from early visual cortex (V2), while the lower traces come from inferotemporal cortex (TE). These can be thought of as first and second level empirical responses respectively. The lower panel (D) reproduces the eye movement trajectories of the previous figure. The simulated electrophysiological responses highlighted in cyan are characterised in more detail in the next figure.

Fig. 7 shows the simulated electrophysiological responses associated with the belief updating reported in Fig. 6. Expectations about the hidden state at the higher (upper panel) and lower (middle panel) levels are presented in raster format. The horizontal axis is time over the entire trial, where each iteration corresponds roughly to 16 ms and the trial lasted for three and half seconds. The vertical axis corresponds to the Bayesian model averages or expectations about the six sentences at the higher level and the three words at the lower level. Under the scheduling used in these simulations, higher level expectations wait until lower-level updates have terminated and, reciprocally, lower-level updates are suspended until belief updating in the higher level has been completed. This means the expectations are sustained at the higher level, while the lower level gathers information. The resulting patterns of firing rate over time show a marked resemblance to pre-saccadic delay period activity in the prefrontal cortex. The insert on the upper right is based upon the empirical results reported in (Funahashi, 2014) and tie in nicely with the putative role of matrix thalamocortical projections during delay period activity (Cruikshank et al., 2012). Note that the expectations are reset at the beginning of each epoch, producing the transients in the lower panel on the left. These fluctuations are the firing rate in the upper panels filtered between 4 Hz and 32 Hz and can be regarded as (band pass filtered) changes in simulated depolarisation. These simulated local field potentials are again remarkably similar to empirical responses. The examples shown in the lower right inset are based on the study of perisaccadic electrophysiological responses in early and inferotemporal cortex during active vision reported in (Purpura et al., 2003).

### 4.1 Summary

In the previous section, we highlighted the biological plausibility of belief updating based upon deep temporal models. In this section, the biological plausibility is further endorsed in terms of canonical electrophysiological phenomena such as perisaccadic delay period firing activity and local field potentials. Furthermore, these simulations have a high degree of face validity in terms of saccadic eye movements during reading (Rayner, 1978, Rayner, 2009). In the final section, we focus on the electrophysiological correlates and try to reproduce classical event related potential phenomena such as the mismatch negativity and other responses to violation.

## 5 Simulations of classical violation responses

**Fig. 8:**
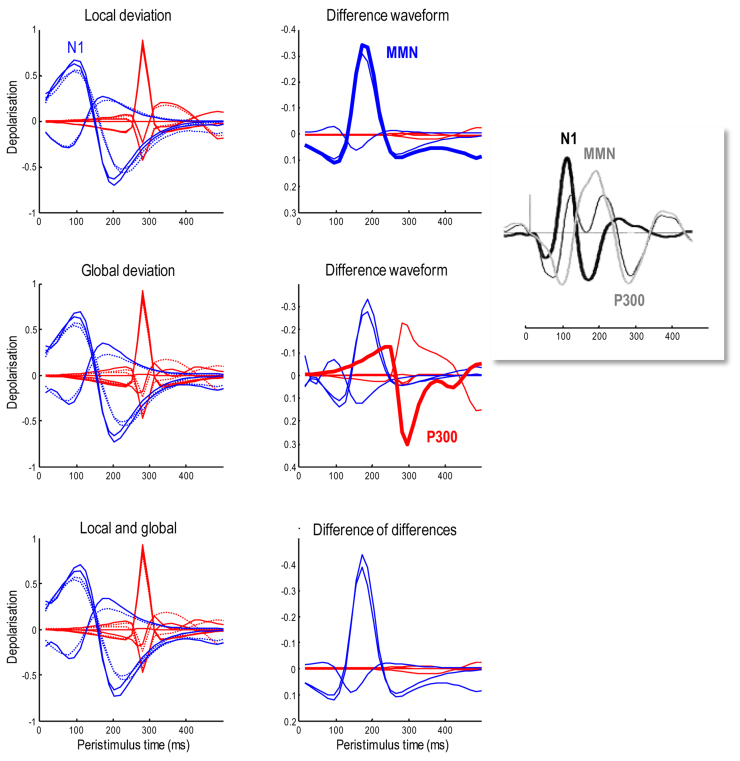
**Simulated electrophysiological responses to violations**: these simulated electrophysiological correlates focus on the perisaccadic responses around the last saccade prior to the final epoch, i.e. the cyan region highlighted in Fig. 7. The blue lines report the (filtered) expectations over peristimulus time at the first level (with one line for each of the three words), while the red lines show the evolution of expectations at the second level (with one line for each of the six sentences). Here, we repeated the simulations using the same stimuli and actions but under different prior beliefs. First, we reversed the prior expectation of a lower case by switching the informative priors on the vertical flip for, and only for, the last word. This means that the stimuli violated local expectations, producing slightly greater excursions in the dynamics of belief updating. These can be seen in slight differences between the normal\ standard response (dotted line) and the response to the surprising letter (solid lines) in the upper left panel. The ensuing difference waveform is shown on the upper right panel and looks remarkably like the classical mismatch negativity. Second, we made the inferred sentence surprising by decreasing its prior probability by a factor of eight. This global violation rendered the sampled word relatively surprising, producing a difference waveform with more protracted dynamics. Again, this is remarkably similar to empirical P300 responses seen with global violations. Finally, we combined the local and global priors to examine the interaction between local and global violations in terms of the difference in difference waveforms (these are not the differences in the difference waveforms above, which are referenced to the same normal response). The results are shown on the lower right and suggest that the effect of a global violation on the effects of a local violation (and *vice versa*) is largely restricted to early responses. The insert illustrates empirical event related potentials to unexpected stimuli elicited in patients with altered levels of consciousness to illustrate the form of empirical MMN and P300 responses (Fischer et al., 2000). (For interpretation of the references to colour in this figure legend, the reader is referred to the web version of this article.)

Fig. 8 shows simulated electrophysiological correlates of perisaccadic responses after the last saccade prior to the decision epoch, when the subject declared her choice (in this case *happy*). To characterise responses to violations of local and global expectations, we repeated the simulations using exactly the same stimuli and actions but under different prior beliefs. Our hope here was to reproduce the classical mismatch negativity (MMN) response to unexpected (local) stimulus features (Strauss et al., 2015) – and a subsequent P300 (or N400) response to semantic (global) violations (Donchin and Coles, 1988). These distinct violation responses are important correlates of attentional processing and, clinically, conscious level and psychopathology (Morlet and Fischer, 2014, Light et al., 2015).

To simulate local (word or lexical) violations, we reversed the prior expectation of an upper case by switching the priors on the vertical flip for, and only for the last word. This produced greater excursions in the dynamics of belief updating. These can be seen as slight differences between the normal response (dotted lines) and response under local violations (solid lines) in the upper left panel of Fig. 8. The lower-level (lexical) expectations are shown in blue, while high-level (contextual) expectations are shown in red. Belief updating at the lower-level produces a fluctuation at around 100 ms known as an N1 response in ERP research. In contrast to these early (a.k.a. exogenous) responses, later (a.k.a. endogenous) responses appear to be dominated by expectations at the higher level. The difference waveforms (with and without surprising stimulus features) are shown on the upper right panel and look remarkably like a classical mismatch negativity. Note that the mismatch negativity peaks at about 170 ms and slightly postdates the N1. Again, this is exactly what is observed empirically; leading to debates about whether the generators of the N1 and MMM are the same or different. These simulations offer a definitive answer: the generators (neuronal encoding of expectations) are exactly the same; however, evidence accumulation is slightly slower when expectations are violated – leading to a protracted difference waveform.

To emulate global violations, we decreased the prior probability of the inferred (first) sentence by a factor of eight. This global (semantic) violation rendered the sampled word relatively surprising, producing a difference waveform with more protracted dynamics. Again, this is remarkably similar to empirical P300 responses seen with contextual violations. It is well known that the amplitude of the P300 component is inversely related to the probability of stimuli (Donchin and Coles, 1988). The anterior P3a is generally evoked by stimuli that deviate from expectations. Indeed, novel stimuli generate a higher-amplitude P3a component than deviant but repeated stimuli. The P3b is a late positive component with a parietal (posterior) distribution seen in oddball paradigms and is thought to represent a context-updating operation (Donchin and Coles, 1988, Morlet and Fischer, 2014). Here, this context is operationalised in terms of changes in (sentence) context, under which lexical features are accumulated.

Finally, we combined the local and global priors to examine the interaction between local and global violations in terms of the difference of difference waveforms. The results are shown on the lower right and suggest that the effect of a global violation on the effects of a local violation (and *vice versa*) look similar to the mismatch negativity. This means, that the effect of a local violation on a global violation is manifest as an increase in the amplitude of mismatch negativity and the positive P300 differences. Interestingly, this interaction appears to be restricted to low (lexical) representations. This suggests that, empirically, a late peak P300 like response to global violations may appear to be generated by sources normally associated with mismatch negativity (e.g., a shift to more anterior sources of the sort that define the P3a).

### 5.1 Summary

The opportunity to simulate these classical waveforms rests upon having a computationally and neurophysiologically plausible process theory that accommodates notions of violations and expectations. Happily, this is exactly the sort of framework offered by active inference. The MMN and P300 are particularly interesting from the point of view of clinical research and computational psychiatry (Montague et al., 2012). Indeed, their use in schizophrenia research (Umbricht and Krljes, 2005, Wang et al., 2014, Light et al., 2015) was a partial motivation for the work reported in this paper.

## 6 Discussion

This paper has introduced the form and variational inversion of deep (hierarchical) temporal models for discrete (Markovian) hidden states and outcomes. This form of modelling has been important in machine learning; e.g. (Nefian et al., 2002, George and Hawkins, 2009), with a special focus on hierarchical or deep architectures (Salakhutdinov et al., 2013, Zorzi et al., 2013, Testolin and Zorzi, 2016). The technical contribution of this work is a formal treatment of discrete time in a hierarchical (nested or deep) setting and a simple set of belief update rules that follow from minimising variational free energy. Furthermore, this minimisation is contextualised within active inference to generate purposeful (epistemic and pragmatic) behaviour based on planning as inference (Botvinick and Toussaint, 2012).

The inference scheme presented here takes a potentially important step in explaining hierarchical temporal behaviour and how it may be orchestrated by the brain. There are a number of directions in which the scope of hierarchical schemes of the sort could be expanded. Firstly, to fully capture the dynamic character of language comprehension and production, means one has to handle systems of compositional recursive rules (Fodor and Pylyshyn, 1988, Pinker and Ullman, 2002), that underlie language grammars (Chomsky, 2006). This is likely to require deeper generative models that may entail some structure learning or nonparametric Bayesian methods (MacKay and Peto, 1995, Goldwater, 2006, Gershman and Niv, 2010, Collins and Frank, 2013). Secondly, there are subtle aspects to processing of serial order that have been identified and modelled (Page and Norris, 1998, Burgess and Hitch, 1999, Brown et al., 2000, Botvinick and Plaut, 2006). For example, without additional mechanisms, associative chaining models – in which chains are constructed with one-to-one dependencies between items – have difficulty modelling repetition (Lashley, 1951). This is because dependencies from an item typically change when it is repeated, requiring context dependent mechanisms to be added. The types-tokens framework (Kanwisher, 1987, Bowman and Wyble, 2007) may be a particularly useful way to handle repetition. In addition to repetitions, error patterns in serial order recall seem inconsistent with a vanilla associative chaining model (Henson, 1998, Page and Norris, 1998). In further work on the deep temporal model presented here, we will explore extensions that enable language grammars and classic serial order recall data to be simulated.

From a neurobiological perspective, the belief updating appears to be sufficiently simple to be biologically plausible; resting on simple operators such as softmax functions, logarithmic transforms and linear algebra (that can be implemented using firing rate functions, nonlinear postsynaptic responses and neuronal connectivity respectively). Furthermore, the intrinsic and extrinsic connectivity required by the belief propagation appears to map gracefully to intrinsic and extrinsic connectivity within canonical microcircuits – and in the cortical-basal ganglia-thalamic loops responsible for action selection in the brain. The computational architecture that emerges from applying standard (variational) Bayesian belief updating to hierarchical models relates observable neuronal dynamics to underlying computational processes; an approach that might be applicable to temporally structured neurophysiological responses across different measurements and cognitive domains (Dehaene-Lambertz et al., 2006, Hasson et al., 2008, Cocchi et al., 2016). Finally, the biological plausibility of the resulting scheme acquires a predictive validity; in the sense that it reproduces some specific violation responses studied in state-of-the-art cognitive neuroscience (Strauss et al., 2015, Uhrig et al., 2016).

## 7 Software note

Although the generative model – specified by the (**A,B,C,D**) matrices – changes from application to application, the belief updates in Fig. 2 are generic and can be implemented using standard routines (here **spm_MDP_VB_X.m**). These routines are available as Matlab code in the SPM academic software: http://www.fil.ion.ucl.ac.uk/spm/. The simulations in this paper can be reproduced (and customised) via a graphical user interface: by typing **DEM** and selecting the **reading** demo.

## Disclosure statement

The authors have no disclosures or conflict of interest.

## Appendix A

These appendices are provided for readers who are interested in technical details. In particular, they provide the derivations behind the (Matlab) implementation of active inference for Markov decision processes used to illustrate reading in this paper. A more detailed description of the update schemes can be found in the annotated (Matlab) routines that are freely available as academic software.

## Appendix A1

*Belief updating*: Bayesian inference is equivalent to maximising the marginal likelihood – or minimising variational free energy – with respect to beliefs (here, expectations) about hidden or latent variables. Free energy is essentially the difference between complexity and accuracy. The free energy for the generative model in Fig. 1 can be expressed as follows (ignoring constants):

(A.1)

The (marginal) free energy of *N* hidden factors at the *i*-th level, under the *π*-th policy, is given by its path integral (see Appendix A2)

(A.2)

Eq. (A.2) expresses free energy as an expected *prediction error*, where the prediction error is the log expectation of the hidden state minus the prediction from its Markov blanket; namely, the previous state, the next state and other factors at the current time (i.e., parents of the current outcome). For the first hidden state, the prediction from the previous state is replaced by the prediction from the hierarchical level above; . For simplicity, we have omitted the time subscript for expected states at the higher level and have assumed a single outcome modality and a single higher-level factor.

The expected free energy of a policy (see Appendix A3) has the same form as (A.2), now allowing for *M* multiple outcome modalities:

(A.3)

The updates for expectations of hidden states can now be formulated as a gradient descent on free energy, while policy expectations minimises free energy explicitly (ignoring constants):

(A.4)

Here,  denotes the expected initial state in the lower level during epoch *τ* at the higher level. Note that the prediction error is simply the free energy gradient with respect to each hidden state. This means that optimising free energy through a gradient descent corresponds to destroying free energy gradients (Tschacher and Haken, 2007).

## Appendix A2

*Marginal free energy lemma:* the variational free energy in (A.2) has been formulated a slightly unusual way to finesse the overconfidence problem that attends mean field approximations. Minimising this marginal free energy, with respect to the approximate posterior, yields the true posterior by the following lemma:

*Lemma* (marginal free energy): under a mean field approximation *Q*(*s*) = ∏_*i*_*Q*^(*i*)^, the marginal free energy **F**^(*i*)^ is minimised when the approximate posteriors becomes the true marginal posteriors, at which point (negative) free energy becomes log evidence:

(A.5)

Proof when the approximate posterior is equal to the true posterior, marginalisation over the Markov blanket of any hidden state returns its true posterior and the divergence attains its minimum of zero

(A.6)

This means the free energy reduces to the log evidence□ Remarks to finesse the problem that we do not have an explicit joint posterior over the Markov blanket, one can replace the complexity based upon the joint posterior with the sum of complexities based upon the marginal posteriors − based upon the assumption that they share the same minimum.

(A.7)

In practice, this appears to offer robust convergence. This leads to the marginal free energy in (A.3) in which the complexity can be separated into prior constraints from the parents and children of states at any point in time (that can be construed of in terms of a forward and backwards complexity).

## Appendix A3

*Expected free energy:* variational free energy is a functional of a distribution over states, given observed outcomes. We can express this as a function of the sufficient statistics of the posterior:

(A.8) *F*(**s**) = *E*_*Q*(*s*|**s**)_[ln*Q*(*s*|**s**) − ln*P*(*o*, *s*)]

In contrast, the expected free energy is the average over (unobserved) outcomes, given some policy that determines the distribution over states. This can be expressed as a function of the policy:

(A.9)

The expected free energy is therefore just the expected entropy or uncertainty about outcomes under a particular policy. Things get more interesting if we express the generative model-terms of a prior over outcomes that does not depend upon the policy

(A.10)

In this setting, the expected uncertainty or ambiguity is supplemented with a risk that scores the difference between the prior (preferred) outcomes and outcomes under a particular policy. This is the form used in active inference, where all the probabilities in (A.10) are conditioned upon past observations. This enables one to replace the posterior in (A.10) with the approximate posterior that minimises variational free energy based on (observed) outcomes in the past.

## Footnotes

1 This is roughly the amount of time taken per iteration on a personal computer − to less than an order of magnitude.
